# State legislation and policies to improve perinatal mental health: a policy review and analysis of the state of Illinois

**DOI:** 10.3389/fpsyt.2024.1347382

**Published:** 2024-04-18

**Authors:** Karen M. Tabb, Wan-Jung Hsieh, Xavier R. Ramirez, Sandra Kopels

**Affiliations:** ^1^ School of Social Work, University of Illinois at Urbana Champaign, Urbana, IL, United States; ^2^ Department of Social Work, National Taiwan University, Taipei, Taiwan

**Keywords:** perinatal mental health, depression, screening, public health policy, mitigating factor

## Abstract

**Introduction:**

Maternal mental health problems, such as perinatal depression, are a major public health issue. In the U.S., several states have policies related to mental health during pregnancy and postpartum. The extent of these laws at the state level needs to be further explored and described.

**Methods:**

We systematically searched the Illinois General Assembly to determine all existing legislation on the topic of perinatal mental health.

**Results:**

This search uncovered two major Acts that 1) require universal perinatal depression screening and 2) raise awareness of the symptoms and treatment options related to maternal mental health. We also discovered provisions in the law that allow for untreated or undiagnosed postpartum depression or psychosis to be considered as a mitigating factor for women who commit forcible felonies.

**Discussion:**

Through legislation, states can lead change at the systems-level to improve perinatal mental health outcomes.

## 1 Introduction

Mental health problems are a serious concern for women who experience a greater burden over the life course, especially during pregnancy and postpartum, the perinatal period. Mental health problems can onset during pregnancy and up to one year postpartum and are often defined as “perinatal mental illness” or “perinatal mood disorders” (1). Some perinatal mental health problems include perinatal depression, generalized anxiety, obsessive-compulsive disorders, panic, social anxiety disorder, and psychosis (2). Depression is common among perinatal women with an estimated one in eight affected by postpartum depression after childbirth (3). Along with perinatal depression, perinatal anxiety disorders are also common among women (2). While not nearly as common, post-partum psychosis is a serious mental illness that affects 1-2 in every 1000 women and occurs rapidly after birth (2). Postpartum psychosis is categorized by symptoms such as hallucinations, mood fluctuation, confusion, delirium, and insomnia (2). Perinatal mental illness can be treated if identified early. If untreated, perinatal mental illness can have adverse effects on perinatal women, newborn infants, and their family members.

Perhaps the most serious result of perinatal mental health problems is the incidence of suicide and filicide among women with postpartum psychosis. Suicide is a leading cause of death among mothers up to one year of delivery. Women who experience postpartum psychosis use more violent means of committing suicide, which significantly differs from the common trend of women committing suicide nonviolently (4). The incidence of filicide after delivery is often, but not always, linked to maternal suicide (5). Neonaticide is a type of filicide in which the mother kills her baby within the first few days of life and infanticide is the killing of a baby within the first year of life (6). The rate of infant homicide was the highest in the United States, occurring in 8 out of 100,000 children (5, 7). Between the years 1976 to 2005, 38.2% of all homicides were children under the age of five that were killed by their mothers (7). While the rate of infanticide has decreased in recent years, infants are 5 times more likely to die by homicide when compared to any other point of the life course (CDC 2020). Accordingly, mental health problems of the mother can potentially present an imminent risk for the offspring.

Universal health screening is a public health approach to detecting mental health disorders for common mental health conditions such as depression or anxiety. Despite its importance, very few states mandate screening for depression or anxiety during pregnancy or the postpartum period. To date, only five states, New Jersey, West Virginia, Massachusetts, California, and Illinois have requirements or mandates for screening for maternal mental health conditions (8, 9). However, of these states, Illinois is the only state with language that involves other state agencies and references the American College of Obstetricians and Gynecology screening procedures as a guide (10). New Jersey and Illinois have the longest standing depression screening mandates and Illinois has continued to develop new policy approaches to improve perinatal mental health. Screening is essential at different points in the perinatal period as it provides insight into developing postpartum depression and/or other pregnancy complications. For many years, the focus of perinatal mental health problems was limited to the postpartum period, but recent trends to screen early and often now capture the antenatal period. People experiencing Antenatal Depression have frequently reported other stressors including conflict with family/partner, risk for miscarriage, and financial difficulties while pregnant (11). Additionally, a recent study found that the presence of maternal blues, which can present between 1 to 10 weeks postpartum, has a significant relationship to higher depressive symptomology scores and the development of depression and/or anxiety disorder (12, 13). Since antenatal mental health problems are often overlooked and represent such a great opportunity for interventions, we want to identify state specific policy levers to improve perinatal mental health. To date, past policy reviews have examined perinatal depression policies broadly across states and have not looked closely at the laws within a particular state that address perinatal mental health (8). Therefore, this policy review conducts an in-depth analysis of Illinois, one of the states that mandates depression screening, to examine its perinatal mental health policies. In this review, we will identify relevant Illinois policies related to the treatment, detection, and handling of perinatal mental health disorders.

## 2 Methods

This policy review study utilizes a qualitative approach to the content analysis used by several qualitative legislative reviews (14). An *a priori* conceptual framework was used to guide the development of the legislative search criteria, the codebook of terms searched, and the analysis of legislation included in the policy review. A priori framework focused on the analysis of policies or programs that had already been passed and were implemented or being implemented. For the systematic search, we used the Illinois General Assembly website (www.ilga.gov), a database that contains a variety of material pertaining to Illinois law. It includes pending bills that have not yet been approved and passed by the legislature, recently enacted public acts (which have passed the legislature and have been signed by the Governor), and most important to this review, the Illinois Compiled Statutes. The Illinois Compiled Statutes is the grouping of all Illinois legislation into an organizational system by general subject and by topics within those subjects. The Legislative Reference Bureau of Illinois organizes all Illinois law into 9 major topic areas which are then divided into 67 separate chapters. Each of the approximately 2000 general Acts of Illinois are placed within one of the chapters in the Illinois Compiled Statutes (15). No Act appears in more than one chapter.

### 2.1 Identifying relevant legislation

We searched the Illinois Compiled Statutes with the goal of identifying all existing Illinois legislation related to perinatal mental health through the end of the 2022 legislative term. Based upon our framework we included the following 12 keywords as search terms: pregnancy, postpartum, antenatal, antepartum, perinatal, postnatal, prenatal, post-partum, puerperium, pregnant, neonatal, and natal. These terms were chosen because they are commonly used to reference the perinatal period. For each search term, we systematically entered the term into the Illinois Compiled Statutes keyword search. We did this separately for each of the 12 terms. When one of the 12 keywords appeared in the keyword search, the chapter in which it was placed, and the section number of the legislation were recorded.

### 2.2 Legislation selection

**Figure 1:**
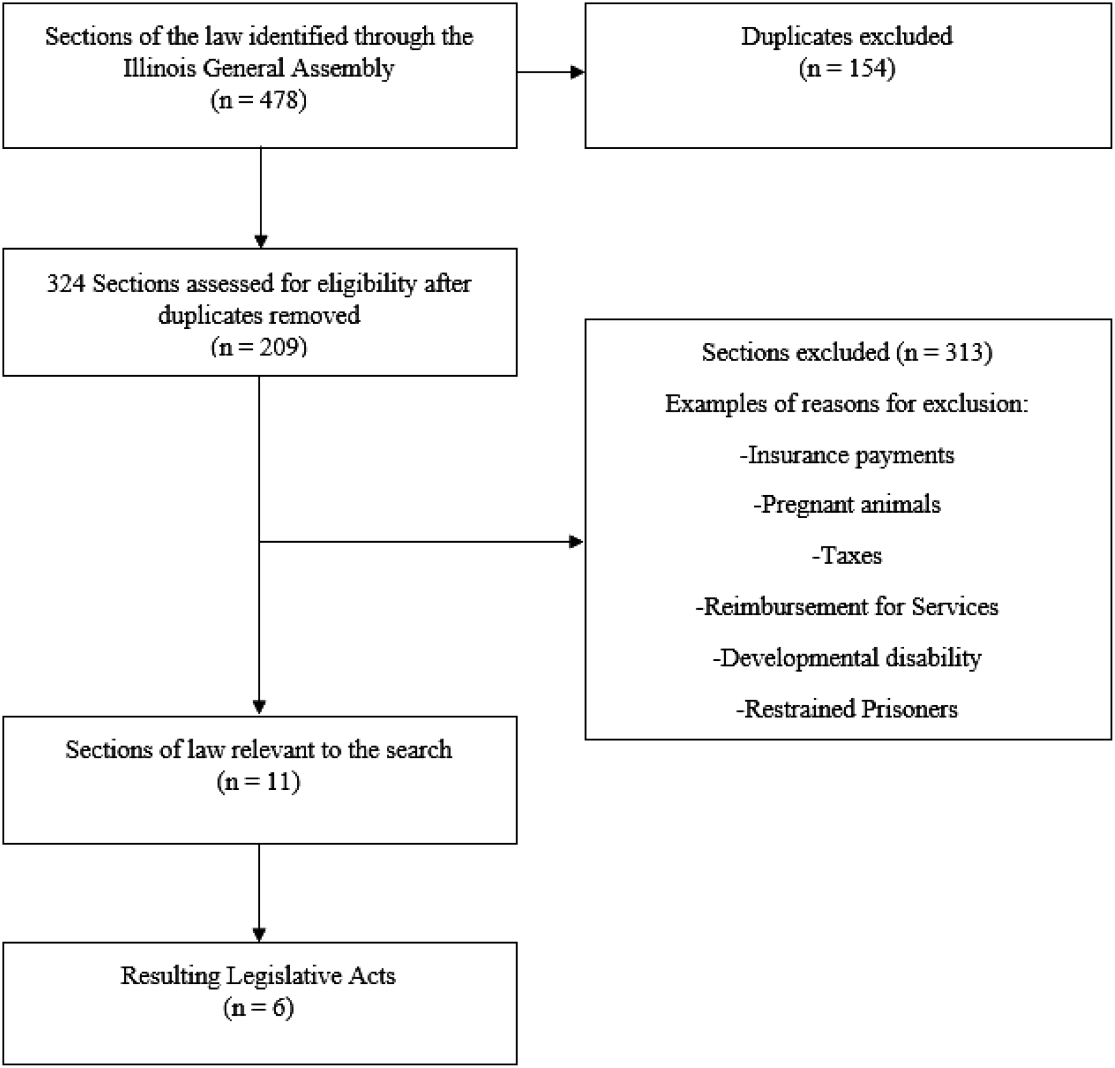
Flow Diagram of Policy Selection and Inclusion in Content Analysis.

As of October 2023, the 12 search terms yielded 478 separate, initial results (see **Figure 1**). Of these 478 initial results, each was read to determine how the search terms were used. 154 results were deemed as duplicates and were removed. A duplicate occurred when more than one keyword appeared in the same section of legislation. For example, within Chapter 325 on early intervention services, the search terms “prenatal,” “perinatal,” and “neonatal” all appeared within the definition of “physical or mental condition which typically results in developmental delay.” One of these results would be kept and the other two would be considered duplicate results and discarded. After the removal of the duplicates, 324 subsequent results were left. The remaining 324 results were examined to determine the subject matter of the legislation. Because the search terms were entered into the database simply as words without any context, the keyword search uncovered that the search terms were used in a variety of topics. Most of these topics were unrelated to the provision of perinatal mental health services. So, for example, the aforementioned result regarding early intervention services would then be removed because the content had no relationship to perinatal mental health. Instead, the result was simply part of the definition of the types of conditions eligible for early intervention services. As another example, the word “pregnant” led to sections on umbilical cord donation after pregnancy, foster care placement of children who were pregnant, graduation incentives for pregnant students, or even prohibitions on restraining pregnant prisoners. In these instances, the term “pregnant” was unrelated to perinatal mental health and was eliminated. As a further example, the word “neonatal” led to provisions in the law regarding taxes for neonatal health services, costs of neonatal home therapy equipment, and data reporting on hospital discharges for neonatal infants. Since none of these cases were related to perinatal mental health, they, too, were eliminated. Therefore, after reading the legislation to ascertain the context for which the search terms appeared in the law, all instances where the search terms were unrelated to perinatal mental health screening and/or pregnant humans were excluded. For the purpose of this policy review, full-text versions of the laws were retrieved.

## 3 Results

The final sample consisted of 11 sections of legislation across six distinct Acts (**Table 1**). The majority of the results were located in two seminal Acts, the Perinatal Mental Health Disorders Prevention and Treatment Act (PMHDPTA) and the Maternal Mental Health Conditions Education, Early Diagnosis and Treatment Act, (MMHCEEDTA). Both Acts comprehensively deal with the prevention, treatment, education, and diagnosis of perinatal mental health conditions.

**Table 1:** Resulting Legislative Acts Related to Perinatal Mental Health Policy in Illinois in 2023.

Civil Administrative Code of Illinois, (2023). Department of Public Health Powers and Duties Law, 20 Illinois Compiled Statutes 2310/2310-223.
Code of Civil Procedure, (2023). 735 Illinois Compiled Statutes 5/2-1401(b-10).
Illinois Public Aid Code, (2023). 305 Illinois Compiled Statutes, 5/5-5.
Maternal Mental Health Conditions Education, Early Diagnosis, and Treatment Act, (MMHCEEDTA, 2023). 405 Illinois Compiled Statutes 120.
Perinatal Mental Health Disorders Prevention and Treatment Act, (PMHDPTA, 2023). 405 Illinois Compiled Statutes 95.
Unified Code of Corrections, (2023). Factors in Mitigation, 730 Illinois Compiled Statutes 5-5-3.1(17).

A smaller subset of results relevant to maternal mental health were also identified in discrete sections of other laws. One result was found in the Unified Code of Corrections, one result was found in the Code of Civil Procedure, one result was found in the powers and duties of the Department of Public Health, and one last result was found in the Illinois Public Aid Code. These results are presented as follows:

The Perinatal Mental Health Disorders Prevention and Treatment Act (PMHDPTA, 2023) was enacted in 2008 and amended in 2015 and 2018. The PMHDPTA contains 4 sections, which include the short title of the law, legislative findings regarding perinatal mental health disorders, definitions of terms, and requirements for perinatal mental health disorders prevention and treatment. In Section 5 of the Act, the Illinois General Assembly lists its findings and the purposes for the Act’s passage. Among the legislative findings and purposes, the Illinois General Assembly found that perinatal mental health disorders include a wide range of emotional, psychological, and physiological reactions to childbirth that can challenge women’s stamina during pregnancy and after birth and impair their ability to function and care for their children. The legislature also found that more than 500,000 women experience perinatal mental health disorders during pregnancy and into their children’s first years of life, annually; women may experience perinatal depression regardless of their previous mental health diagnoses; women suffering from perinatal mental health disorders may require counseling and treatment yet be unaware of the need for or availability of such services; and that not only the women, but also their babies, the fathers, other children, and family members may be impacted by perinatal mental health disorders (PMHDPTA, 2023). Accordingly, the PMHDPTA’s purpose is to provide information to women and their families about perinatal mental health disorders, to develop procedures to assess women for perinatal mental health disorders during prenatal and postnatal visits, and to promote the early detection of perinatal mental health disorders.

Section 10 of the PMHDPTA provides definitions for certain terms used in this law. Interestingly, the word “perinatal” which appears 19 times in this Act, is not defined. The closest the legislature comes to defining “perinatal” is by including the word as part of the term “perinatal mental health disorders” which it refers to as “postpartum depression,” and is also undefined.

Section 15 of the PMHDPTA directs Illinois healthcare agencies to work with hospitals and healthcare providers to develop policies, procedures, information, and educational materials to meet certain requirements. Hospitals that provide labor and delivery services are required to inform mothers, after childbirth and before discharge, and fathers and family members, if possible, about the symptoms of perinatal mental health disorders, coping methods, and resources for treatment. Healthcare professionals who provide prenatal services must provide education to women, and their families, if possible, about perinatal mental health disorders. At prenatal visits, healthcare professionals must invite patients to complete questionnaires and review them following the recommendations of the American College of Obstetricians and Gynecologists (*Patient Screening*, n.d.). When the professional judgment of the healthcare professional indicates that a woman may be experiencing a perinatal mental health disorder, the healthcare professional must complete additional assessments. When healthcare professionals provide postnatal services, they must also invite women to complete questionnaires. Additionally, healthcare professionals who provide pediatric care to infants shall invite mothers to complete questionnaires at well-baby check-ups before the infants’ first birthday to ensure that the infants are not compromised by the mothers’ undiagnosed perinatal mental health disorders.

The Maternal Mental Health Conditions Education, Early Diagnosis, and Treatment Act (MMHCEEDTA, 2023) became law in Illinois in 2020. This Act includes the short title of the law; legislative findings regarding maternal mental health conditions; definitions; and educational material about maternal mental health conditions. In Section 10 of this law, maternal mental health conditions are defined as mental health conditions that occur during pregnancy or during the postpartum period, including, but not limited to postpartum depression. In Section 5 of the law, the legislature found that maternal depression is a common complication of pregnancy and that maternal mental health disorders include depression, anxiety, and postpartum psychosis. The Illinois General Assembly noted that maternal mental health conditions affect one in 5 women during or after pregnancy but that all women are at risk of suffering from these conditions. The legislature also found that untreated maternal mental health conditions have short-and long-term negative impacts on the health and well-being of affected women and their children: these impacts cause adverse birth outcomes, and emotional and behavioral problems in childhood and have significant medical and economic costs. The legislature estimated these costs to be $22,500 per mother. By passing this Act, the legislature stated its intention to raise awareness of the risk factors, signs, symptoms, and treatment options for maternal mental health conditions.

In Section 15 of the MMHCEEDTA legislation, the Illinois Department of Human Services (IDHS) will develop educational materials both for health care professionals and patients. Obstetric units will take the educational materials and provide them to their employees who regularly work in patient care for pregnant and/or postpartum women. Additionally, hospitals will incorporate the information into employee training and augment the information with relevant local resources. The information IDHS develops for postpartum women and their families will educate them about maternal mental health conditions, treatment options post-hospitalization, and community resources.

Four other results relevant to maternal mental health were also identified in discrete sections of other legislation. Illinois allows maternal mental health to be considered as a factor in sentencing for crimes or in seeking relief after already being convicted of a crime. In the Unified Code of Corrections, post-partum depression or post-partum psychosis are factors in mitigation that may be considered when withholding or minimizing a defendant’s prison sentence (Unified Code of Corrections, 2023). To be used as a mitigating factor, the post-partum depression or post-partum psychosis must be undiagnosed and/or untreated at the time of the offense; the condition would tend to excuse or justify the defendant’s criminal conduct; a qualified medical person has since diagnosed the defendant as having suffered from these conditions; and the diagnosis or testimony about the illness was not used at the trial. In this law, post-partum depression is defined as a mood disorder that occurs during pregnancy and up to 12 months after delivery and can include anxiety disorders. Post-partum psychosis is defined as an extreme form of post-partum depression that can include “losing touch with reality, distorted thinking, delusions, auditory and visual hallucinations, paranoia, hyperactivity and rapid speech, or mania” (Unified Code of Corrections, 2023).

Similarly, the Code of Civil Procedure, (2023), allows a defendant to file a petition for post-judgment relief if she can establish that her participation in a forcible felony was a direct result of suffering from post-partum depression or post-partum psychosis. A defendant needs to demonstrate that there was no evidence of post-partum depression or post-partum psychosis presented by a qualified medical person at trial and/or sentencing. A defendant must demonstrate that she was unaware of the mitigating nature of her post-partum depression or post-partum psychosis or that if she was aware, she was unable to present this defense. Her inability to present the defense must stem from her suffering from these conditions at the time of her trial or sentencing. Alternatively, a defendant can establish that the post-partum depression or post-partum psychosis were not recognized mental illnesses at the time of the trial or sentencing and because of that, she was unable to receive proper treatment. She must also show that the evidence is of such a conclusive character that it would likely change the sentence that was imposed by the original court. In this law, post-partum depression and post-partum psychosis are defined exactly as they are in the Unified Code of Corrections.

New Illinois legislation directs the Illinois Department of Public Health (IDPH) to establish a classification system for levels of maternal care, including basic, specialty, subspecialty, and regional prenatal health care (Civil Administrative Code, 2023). One of the legislative directives requires IDPH to engage the Illinois Chapter of the American Academy of Pediatrics to expand efforts so that physicians conduct postpartum depression screenings at well-baby visits during the child’s first year of life.

The final result of the content analysis uncovered a provision in the Illinois Public Aid Code. The Medical Assistance Program provides essential medical care and rehabilitative services to persons who are financially unable to meet their basic medical needs. As of July 2022, coverage for perinatal depression screenings for the 12-month period after pregnant persons give birth is an eligible and covered service (Illinois Public Aid Code, 2023).

## 4 Discussion

This policy review sought to identify relevant policies to address maternal mental health in Illinois. Eleven unique sections of legislation were uncovered concerning perinatal mental health within six different Acts enacted since 2008. This policy review of a single state finds that there is an increase in perinatal mental health legislation which aligns with previous reviews of legislation across the United States. In their 2013 review of legislation on perinatal mental health, Rhodes and Segre documented Illinois as one state with at least one Act related to perinatal mental health (10). As a contribution, this study provides specificity by systematically reviewing all legislation in one state and conducting a deep level policy content analysis. From this review, we were able to identify the many sections across statutes that relate to perinatal mental health. Of the six Acts, there are exceptional examples that capture Illinois’ effort to address perinatal mental health. The first example is from the PMHDPTA which is similar to other states, that mandates universal depression assessments during pregnancy and postpartum. Perinatal mood disorders such as perinatal depression and anxiety are largely undetected in obstetric care. Even when they are detected, they are left untreated, as only 22% of women who screen positive for depression receive mental health services (13–15). Early detection of untreated mental health problems could be an essential step in preventing serious acts of harm to oneself or others. While it is difficult to assess the rates of perinatal anxiety and psychosis by state, among the 27 states that collected Pregnancy Risk Assessment Monitoring System data in 2012, Illinois had the second lowest rate of postpartum depressive symptoms at 8.1% (16). Within Illinois, past studies found that the rates of depressive symptoms and suicidal ideation detected on depression screens can be higher for low-income populations and racially minoritized populations (17, 18). Mandates for depression screening can be effective to motivate clinicians and health care systems to adopt screening practices (19, 20). Screening remains an essential approach to identify those in need of mental health care (21). Early detection of untreated mental health problems could be essential in preventing serious acts of harm to oneself and/or others.

The second example is the Maternal Mental Health Conditions Education, Early Diagnosis, and Treatment Act. Illinois’ recognition that untreated mental health conditions negatively impact the short-and long-term health and well-being of pregnant persons and their children and require awareness and education regarding these conditions. Often providers are unaware of how to identify and treat mental health problems widening the gap of untreated mental health needs. Untreated perinatal mental health problems are common and costly. Policy level interventions offer unparalleled opportunity to create systems level approaches to identifying those in need of care along the perinatal mental health treatment pathway (22, 23). In some states, legislative decisions have resulted in psychiatry access programs to assist providers in treating mental health problems. Access programs have been used with children and adults and offer promises to address unmet patient needs to achieve equity in perinatal mental health outcomes (24). Access programs are relatively new with the number of states adopting these programs at a swift pace (24). Now that nearly half of the states have perinatal psychiatry access programs, data collection efforts are needed to identify the effectiveness of these programs and any improved access to treatment (24).

The final example is the Unified Code of Corrections. Illinois is the first state to pass a law that allows postpartum depression and psychosis to be used as a mitigating factor in the sentencing of crimes. The Act allows postpartum depression or postpartum psychosis to be considered as a mitigating factor in forcible felonies committed by women who were neither treated for nor provided evidence of postpartum depression or postpartum psychosis at their trials or sentencing. Much earlier papers, such as Kendell et al. (1987), found women in the postpartum period were 30 times more likely to have a psychiatric admission than in any point in life (25). However, many women are undiagnosed and do not receive the treatment that they need (17, 18). This legislation takes the possibility of a misdiagnosis or underdiagnosis into account. This law recognizes that women may have undiagnosed or untreated mental health conditions related to their pregnancy and childbirth, which, in turn, may have contributed to their criminal behavior. Accordingly, the passage of the act creates a need for forensic review of previous cases to establish if undiagnosed or misdiagnosed mental health problems were related (26). Significantly, the law provides definitions of post-partum depression and post-partum psychosis and defines these conditions as occurring during pregnancy and up to 12 months after delivery. Following the passage of the act, over 20 women were among those who could possibly have a reduced sentence (27). Advocates from Illinois are working to use the example of Illinois to shift national policy and to bring awareness of maternal mental illness into law and corrections (28).

In addition to influencing state-level policy, legislators from Illinois also have been responsible for introducing several pieces of significant legislation to address perinatal disorders at the federal level. For example, U.S. Representative Bobby Rush and U.S. Senator Dick Durbin co-sponsored the Moms Opportunity to Access Help, Education, Research and Support for Postpartum Depression Act, or MOTHERS Act. Language from the MOTHERS Act can now be found in Section 2952 of the Patient Protection and Affordable Care Act, which expanded funding for research and programs on perinatal depression and psychosis. Additionally, Illinois was the first state to expand the provision of Medicaid from 60 days to the first year after giving birth (*Pritzker Administration Announces Illinois Is First State to Extend Full Medicaid Benefits to Mothers 12 Months Postpartum*, n.d.). The expansion of Medicaid should increase opportunities for access to providers and reduce risks of complications due to untreated mental health problems. However, data is needed to support the impact of Medicaid expansion. This study presents a strength of closely examining legislation in a single state to determine the state’s efforts to address perinatal mental health, while past studies have broadly compared all states in the US. However, not one study, including ours, has compared legislative acts within and across states. Examining specific policies within states and comparative analyses across states are needed to determine policy implementation effectiveness (24). Future studies are needed to carefully contrast legislation relevant to perinatal mental health at the level of state policy making.

## 5 Conclusion

From this policy review, Illinois has emerged as a leader in passing legislation to address the detection of perinatal mental health problems and the ramifications of untreated mental health problems. Foremost, the PMHDPTA mandates universal depression screening during pregnancy and postpartum and the MMHCEEDTA raises awareness of risk factors, signs, and treatment options related to maternal mental health conditions. More recently, legislation allows untreated or undiagnosed postpartum depression or psychosis to be used in the defense of criminal cases. This significant legislation provides a pathway for mental health to be considered in cases of infanticide and is the first of its kind to pass in the United States. It will be important to review the impact of these policies on national adoption of legislation to address maternal mental illness (28). While Illinois is a leader in advancing perinatal mental health legislation in its state, there remains a need for further reform in all states. From this policy review it is clear that one state in a high-income country is making strides to intervene in untreated mental health problems through legislation, but comparisons are needed across low- and middle-income country settings as well. Future policy reforms are still necessary to improve barriers in health services delivery and to improve perinatal mental health outcomes in high income countries.

## Data availability statement

The original contributions presented in the study are included in the article/supplementary materials. Further inquiries can be directed to the corresponding author.

## Author contributions

KT: Conceptualization, Data curation, Formal analysis, Methodology, Supervision, Writing – original draft, Writing – review & editing. W-JH: Data curation, Formal analysis, Methodology, Writing – review & editing. XR: Methodology, Writing – review & editing. SK: Conceptualization, Data curation, Formal analysis, Investigation, Methodology, Supervision, Writing – original draft, Writing – review & editing.
